# Supplementary material for: Genome-Wide Transcriptomic and Proteomic Exploration of Molecular Regulations in Quinoa Responses to Ethylene and Salt Stress
Source: Plants (Basel). 2021 Oct 25;10(11):2281. doi: 10.3390/plants10112281 (PMC8625574; doi:10.3390/plants10112281)
Supplement: Supplementary file 1 [file plants-10-02281-s001.zip › plants-1403961/SUPPLEMENTARY MATERIALS/SUPPLEMENTARY MATERIAL TABLE S1-QIAN MA.pdf]

>110717159 (*CqGLC*)
ACCTTTGCATGATAATGTAATCAACAATTAAAACTATTAAACTTTGTTCTTAA
GTAAATTCAATCTCAAATGGGGTTAATAAGCAAACCTTCTGCGGTTGCTACT
TTGCTGCTTCTTGCAACATTTCTACCTAGTCTTCAAGTAACAGAGGCGCAAA
TTGGTGTATGTTATGGAAGAAATGGGGACAATCTACCATCCCAACAAGAAG
TGGTTACGATGTACAAAAATAATGGAATAACACGTATGAGACTATATGATCC
CGACCAAGGATCCCTCCAAGCCCTTAGAAATTCAAACATAGGGCTAATCCT
TGATGTCCCTACCGACAAAGTCATCTCACTCGCGAATGCTGCAAATGCAAG
GACATGGGTCCAAAACAATGTAGTCCCTTACGCGTCTAATGTGAATTTTCGT
TACATTTCAGTAGGTAACGAAATCATGCCTGGTGAGGCCGCGGCAGGATCG
GTCTTACCTGCCATGCAAAATGTCCAAAATGCCCTTAATGCAGCTAACTTAG
GTGGTAAAATCAAAGTCTCCACAGCAATCAAGAGTCAGATTGTTTCGGGTT
TCCCACCTTCTGCCGGTGTTTTTACATCTTCACAATACATGAATCCAATTGTT
AACTTTCTAAAGAGTAACAATGCTCCTTTATTAGCCAATATCTATCCATACTT
CTCTTACCTTGGTACTGATTCTATAAAACTAGATTACGCGCTCTTTACTTCAC
CCAACGTCCAAGTTACTGATCCAAATAACGGTTTGAAGTACCAAAACCTAT
TTGATGCACTAGTGGACACGGTGTATGCTGCATTGGCAAAGGCTGGAGGCC
CTAACGTACCAATTGTCGTGTCCGAAAGCGGGTGGCCTTCGGCTGGTGGTG
ATAGACGTGGTGCTGCTACCTTTGGTAACGCAGGACAGTATTACAAGGGTT
TAATAGGCCATTACAAGCAAGGGACTCCCTTGAAGAAAGGAGCAATTGAG
ACGTATTTATTTGCTATGTTTGATGAGAACAGAAAGAGTGGTGGTACTGAG
AATAATTTTGGACTGTTTAGGCCCAATAAGCAGCCCAAATACCAACTCAGTT
TCAATTGAAATTTAATTACTCGTATATAGTTTTCTGATATACTTCGTTCGTATA
ATCGTATGATGTTATAATCATCATAATGAATAAGTGAAATAATGTTACACAGG
CTTTGCCTAAATATGTTATAAGTGTTATATACTTTTACTTGTAATAATGTTATT
GATATTATGCCCGTCCAAAAAAAGTACTCCGTATTATTGAAATATTATGCACT
A

>110722212 (*CqABCB*)
TTGTCTTTTTCACAAACTCCATACGTTGGCAATGTCTTTGTTCAACTTTACA
GCAACCCACCAAAATTGGCACTAATTTTACAGTATTATTACTAGTTTAGTTAT
AGGCCTCCCAAAGTCCCAAAGCCCAATATCAAATCTGCTACttctatttataatctcata
TTCAACACTAACATCTAACCACTTGTTTCTTGTTCATTGATTTTTCAACATTG
ACAACATTTTGTAATTTTCTTAAGATTTTTTTAGGATAAATTTGAGTTGGGTT
TACAAGAAAAGGCATGAATTTTGGAGGGCATGGTGGTGGTGGTGGTGGTG
GGTATGGTGGTGGTGGACATGGTTTAGGCGGTGGTGGCGGCGGTAATAGAG
TTCCTCTTTTGGGTCGGCATGCCGGCTTCAAAAGAGAGGAAATTGGCTCAA
CAGACCTTGAGAATGGTAATGCACCGGCTGCAAATGTGGGATTCGGTAGGG
TTCTTTCTCTTGCAAAACCTGATGCACCTAAACTTGTTATAGCTACAGTTGC
TCTGTTGATTGCAGCCACATCAAACATATTAGTGCCAAAGTTTGGTGGAAA
AGTAATTGATATTGTGTCCGGAGATATCAGCTCACCAGAAAAGAAAGCTGA
AGCTCGGGATGCAGTTACGAGCACCATACTAGAGATTGTCATGGTTGTTATT
GTAGGTTCAATTTGCACAGCATTGAGGGCGTGGCTATTTAATTCTGCAAGTG
AAAGGGTTGTTGCAAGACTGAGAAAGAATTTATTCTCCCATCTAATTTGCC

AGGAAATTGCTTTTTTTGACGTTACTCGAACTGGAGAGCTTTTGAGTAGAC
TAGCTGAAGACACACAAATCATAAAGAATGCTGCTACTACTAATCTGTCTG
AAGCTCTGAGAAATTTAGCAACTGCATTCATTGGTCTTGGATTCATGTTTGC
AACATCATGGAAGTTGACATTGTTGGCATTGGCTGTTGTGCCTCCCATTTCT
GTTGGTGTGCGTAAATTTGGTCGCTTTCTCCGTGAACTCTCACACAAGACT
CAGGCTGCTGCTGCTGCAGCATCTTCAATTGCAGAGGAATCGTTTGGTGCC
ATCCGCACAGTAAGGTCCTTTGCCCAAGAAGATTATGAAATTCAGCGGTATT
CTGAGAAAGTTGACGAGACTTTAAACTTGGGACTTACACAAGCTAAAGTT
GCAGGTTTATTCTTTGGAGGACTAAATGCAGCATCCACCCTGTCAGTTATTG
TTGTGGTGATATATGGAGCTTTCCTGGCTATCAATGGTACTGGCATGACACC
TGGTGCTCTCACATCCTTCATACTTTATAGCCTTACAGTTGGGTCATCCATAT
CTGGACTTTCTGGATTATACACTGTGGCAATGAAAGCTGCGGGAGCCAGTA
GGCGTGTTTTCCAACTGCTAGACCGAACATCATCCATGCCAGAGCCAGGAT
CCAAGTGTCCATTAGGTGATCAAGATGCAGAAGTGGAATTGGATGATGTCT
GGTTTGCATATCCTTCACGCCCAAGTCATATGGTACTAAAGGGAATAACGTT
AAAACTGCTGCCTAGTTCAAAAGTTGCACTTGTTGGTCCAAGTGGTGGTGG
AAAAACTACGATAGCACATTTGATTGAAAGATTTTATGATCCTACCAAAGGG
AAGATTTTGCTGAGTGGGGTTCCGCTAGTAGAAATATCTCACAAGCACTTG
CATAGCAAGATTAGTATTGTAAGCCAGGAGCCAACTCTCTTCAATTGCTCCA
TAGAAGAGAACATCGCTTATGGACTAAATGGCAAGGCCAGCACTGCTGATA
TAGAAAATGCTGCTAAAATGGCAAATGCACATGAATTTATATCCAAGTTTCC
TGAGAAGTATCAAACTCATGTCGGAGAACGTGGGGTTAGATTATCAGGTGG
TCAGAAACAGCGGATAGCAATTGCCAGAGCTCTGTTGATGAACCCAAAAG
TACTACTTTTGGATGAAGCAACGAGTGCCCTTGATGCTGAAAGCGAATACT
TAGTGCagGATGCCATGGATCGCTTGATGAATGGAAGAACTGTCCTCGTGAT
AGCACACAGGCTTTCCACTGTGAAAAGTGCAAATGTTGTAGCTGTTATATC
CGATGGTCAGGTGGTGGAAAGCGGCACTCATGATGAACTTTTGAGCAAGG
ATGGCATCTACACTGCACTAGTGAGGCGTCAGTTGCAAGGACCCAAAGCC
GATAGCTAACATTGGAGATATTACTTTACTTACAATTCAGAATACAATTTCAG
AATTTCTATTTGTATTATAGGTCTTCTCTGCCATGTAACTGAACAAGTGAAGC
TTAATTTTTTGGCTTCCGCGTCTCTGTATGTCTTTATTGGGTCTATTAAAGGA
ATGTAAGGTCACCTACTCACCTTAGTCTTTAATCAAGTATTTTATCTGCATAA
TCTGTAAACTACTTCTGTTTTGCTTCACTAATATTATCTTAATTTTGATGGGG
GCAATCCCAATGTC

>110688100 (*CqNRT2.1*)
ACCCTTTTAAAATTATTATTAATTAAATATCATATGATTACGTAATGTACTCGT
TGGGATCTTTACTGGCTTTATTATCAACTTTTATAATGTCAACGTTACCTTACT
ATAAATATTCATGAATACAACAACGTAATACAACAACAAGCCTAATACAACT
TTTTCTTCTTGTACTAGTGTACAACATTTAGATTTTTCAAAATCCCTAAAACA
AAAAACAAAAAAACATGGCCGGTGAGCCTGGTAGCTCCATGCATGGAGTC
ACCGGAAGGGAACAATCGTACGCCTTCTCGGTCTCCCCCTCCACGGTCCCA
ATAGACCCGACGGCCAAGTTTTCTTTGCCCGTCGATTCAGAGCACAAGGCC

ACAAGGCTTAAGATATTCTCCTTGGCAAAGCCTCACATGACCACATTCCAC
CTTAGTTGGATATCATTTTGTGCGTGTTACATATCCACTTTCGCGGCTGCTCC
CTTGGTTCCTATTATTAGGGACAACCTTGACCTTACTAAGAGGGATATTGGT
AATGCTTCGGTTGCATCCGTCTCAGGAAGTGTCTTCTCTAGGCTAGTCATGG
GAACCGTGTGTGACCTCATGGGACCAAGGTACGCTTGTGCGTTCTTAGTCA
TGTTGACCGCCCCTACGGTCTTCTGCATGTCGGTTATAGACGATGCAGCTGG
ATACGTGGCGGTGAGGTTTATGATAGGTTTTTGTCTAGCCACTTTCGTGTCG
TGCCAATATTGGATGAGTACCATGTTTAATGGACGGATCATTGGGTTGGTTA
ATGGAACGGCGGCAGGGTGGGGAAATGTGGGAGGCGGTGCAACCCAACT
TCTTATGCCTTTGGTTTTCGAGATCATCCGGCGTGCAGGAGCTACACCATTT
ATAGCATGGAGGATTGCCTTCTTTCTACCAGGAATGTTACATATTATTATGGG
TATTTTGGTGCTAGCACTTGGTCAAGACTTGCCTGATGGTAACCTTGGTGCC
TTACAAAAAAAGGGTGAGGTTGCCAAAGATAAATTCTCTAAGGTACTTTGG
TATGCAATATCCAACTACCGTACATGGATCTTTGTGCTCCTCTATGGCTATTC
TTTGGGTGTGGAGCTAACAACTGATAATGTCATTGCAGAATACTTTTTTGAC
AGATTCAACCTCAAGTTGCACACAGCAGGAATCATCGCAGCTAGTTTTGGA
ATGGCTAACTTATTTTCCCGCCCCTTAGGAGGGCTAGCTTCCGACTACAGTG
CACGTTACTTCGGTATGAGAGGCCGACTTTGGACCCTTTGGACCTTCCAGA
CCTTGGGTGGTCTCTTCTGCGTATGGCTAGGCTTAGCCAAGACCCTACCCA
CTGCCATCGTAGCACTAGTCCTTTTCTCACTAGGGGCTCAAGCCGCGTGTG
GGGCAACCTATGGGGTCGTCCCCTTCATATCTCGACGATCCCTAGGGATCAT
CTCGGGCCTCACGGGTGCAGGAGGCAACATTGGATCCGGATTGACTCAATT
TATATTTTTCACCGCGGGTAGTTTTAGCACTCACCGAGGTCTTACCTACATG
GGAATCATGACTATTGCGTGCACATTGCCCGTGACCTTGGTGCATTTTCCGC
AATGGGGTAGTATGTTCTTACCCCCTACTAAGAATGCTAATGAGGAGGCTTA
CTATGTTGCTGAGTACGACGACGACGAGAAGAGAAAGGGAATGCATGAAG
GAAGTGTCAAGTTTGCTGAAAGTGCCCGATCTGAGCGAGGTGGGAAGTCT
GCTGCGGCTACCCCTACACAACGTCCCAACCCTAACAATATTTAATAAGTTA
CTTTCGTATTTTATTTGCTTTTGGCTTAAACAACAATGATATGAGATTATGAT
GAACTCCTGTAGTAAATTATTGGAGTTTGTTTTATTTTTATGTTTGGTTTCTTT
TTTTTATATATATCATATATACTTTTGGATTTTTGGCTCAAGAGTTATGGAGAT
AACCTTGTAGCAAGTGAATTTTGCTTGCAATAACTTACATACATGTATACTAT
TACTTCTGTATCTTCAACAGTTCTTGCCAACTTTAATTTAATTTAGAGGTCTT
TCCTAGCCTTATAATGGCTAAGGTATGTGCCTCTTTAATCTTGTTTTTTTTGTT
CAA

>110711362 (*CqAOBG*)
ATTACAAATAACGGGTCCAAGTCAGGTTGATATGTACACGCACTAAAGTTT
GTAAAGAAAAAAAACACATGGAAGAAAATGGAAGCAAAAAAAGCAATCT
AACAGCGTACTTTATCCCGTACTTGACACCGAGTCACTACTTACCTCTCTTC
CAAATTGCTAAGTTGTTTGCATCCCGCGGGGTGCACGTAACATTCCTTACCA
CTTATCACAACTCCCTCTCTTTTCGCGATTCAATCGATTCCCTCAACAAACT
CGGCCTTGACATCGACCTCGACTATGTTCACTTCCCTAGCAAGGAAGTCGG
CTTGCCCGAGGGGGTTGAGAACTTCAGTGCCTCTCCTAACTTGGAAATTGC

CGGTAAAATCTTCCGAGCTTTTTTCATGCTTCAAGCTCCCATCGAAGCTAAG
GTTCGGGCTGCTAAGCCTGACTGTATTGTCGCTGATATGCACTGTTTTTGGA
CCACTGAACTCGCTGCCAACCTTGGTATCCCTCGTTTGATTTACCATGTTCG
AGCTGCTTTTGCCCTCTCAGCTGTTGATGCCATTGATAGGTATACTCCTTATG
AGGATGTGAAATCTGACGACGAAACCTTCCTACTACCGGGACTCCCACATC
CAATATACATGACCCGCTCAGAACTTCCGCAATGGATCCAAACTCCGAGTC
CCTACACCACATTTTCCAACAAAGTTAAAGAGGCTGATCGCAACTGTTATG
GTGTTCTTGTTGATAGCTTCTATGAGTTGGAGAAAGACTACATAGATTACTT
CCGGAACACCTTGGAACGTCGTACTTGGTGTATCGGCCCTCTTTTCCTACAC
CATGATCTTGTTCTCAAAAAACTCAACTCCGGCTTGGACAACAACAACAGC
AAAGCAGAGGAGACCGATAAACACCCTTGTTTGGAATGGCTGGATAAAAT
GCCACAAGGAAAAGTTGTGTATGTAAGCTTTGGAAGCATATCAAGGTTTAG
CTCAGCTCAGCTGTTTGAGATTGCTGAGGGACTTGAATCTTCAGGCCAACC
ATTTATTTGGGTGGTCCGAAAAACGGAGGGGGAGTCCAAGGAAGTGACGG
ATAAATGGCTACCCGAGGGATTTGAGGAAAGAATTTCTGAGAAAAGTATTG
GGATGCTTATACAAGGATGGGCACCCCAATTAAAGATATTGGAGCACCCGG
CCATTGGTGGGTTCGTGACTCACTGTGGGTGGAACTCATCGGTAGAGTCCC
TTTCCGCGGGGCTACCTGTGGTGGCCTGGCCTCTTTCTGCTGAACAGTTCTA
TCATCGAAAGTTATTTATCGATGTATTAAAGATCGGAGTTGGGGTTGGGAAT
ATGAAGTGGAGCTCCATGATTGATGGAACGGATGAGTTAGTGAAGAGAGAT
AAGATTGAGAATGCTGTGAGGGAGTTGATGGGAGATCATGAGGAGGCTCA
GGAAAGGAGGCAGCGCGCCAAGGAGTTTGGCATCGCCGCTAACAAGGCTG
TTCAACAAGGTGGTTCTTCTTATGATGACTTAACTGCTGTCATTGATGAACT
ACAACGTCTCAAGACATCAAATGATGTTGCATGATTCCAACGTCTCAAGAC
ATCAAGTGTGAAGCAAGTTAAAATTGTGCTCAATTATATGCACAAATTAAAT
TTAAATAATTATGATTATGGAAATTGTGCTTGTTTTGATTTTTAAGGGCT

>110717430 (*CqCSI*)
ATATTTGGTTCTTAATCGTCAGTTTTATAAATACTAAAATTACTAGCCGAGTA
AGAACTCTCATGCCATTTCGAAATGGCGGCAACACACATTTGCAAAGTCCA
AACCAAAAGAGCCATTTTTAACCGTATTCACATCCTCATACACTCCTTTGCC
ATTCTTGCTCTCTTCTACTACCGTTTTTCGTCTTTCTCCAACCCTCGTATCTC
CCTTCTGCCATGGATCTTATTGACCATCGCCGACCTCGTTTTCACCTTCGTTT
GGGCCATGACTCAGGCCTTCCGTTGGCGCCCCGTCTTGCATGACGTGTCTG
GCTATGAGTCCATCAATCCACGTGACCTTCCAAGGATCGACGTTTTTATATG
CACCGCCGATCCTACCAAGGAGCCTGTGTTGGAAGTGATGAACTCTGTGAT
ATCATCCATGGCGCTCGATTATCCGCCTGAAAAGATGGCGATATATTTGTCG
GATGATGGAGGTTCTCCTTTGACTAGAGAGGCTATTAAGAAGGCTGTTGAT
TTTGCTAAGGTTTGGATTCCTTTTTGTAACAAGTATGCTATTAAGACTAGGT
GTCCAGATGCTTTCTTCTCCGCTTTGGGTGATGATGAAAGACTTCATTGGGA
TCACGACTTTATCGCTCATGAATCACTGCTAAAGTCGGAATATGAAGCTTTT
AAGAAATATGTGGAGAAAGAAAGCGGTGATTATAATAAATGCACCGTTGTG
CATGATCGTGCCCCTTGCATCGAGATTATACATGACAGCAAGCAGGATGGA
GAAGGTGAAGTGAAAATGCCCCTTGTGGTTTATGTAGCCAGGGAAAAGAG

ACCAGGTCTTCCTCATCGTTTCAAAGCTGGAGCCCTTAACGCTCTTCTTCG
AGTATCAGGTTTATTGAGCAATGCGCCTTACTTATTGGTGTTGGATTGTGATA
TGTACTGTCATGATCCAACCTCTGCTCGTCAATCTATGTGCTTCCATCTTGAC
CCAAACATGGCTCCCTCTCTTGCCTTTGTTCAATACCCGCAAATTTTCTACA
ACACTAGCAAAAATGATATCTATGATGGCCAAGCCAGATCAGCTCATACGA
CAAAATGGCAAGGCATGGATGGACTCAGAGGACCGGTCTTGAATGGAACT
GGGTATTATCTGAAGAAGAAGGCAATATATGGAAGGCCTCATAATGAAGAT
GAATACCTCATCAATGAACCAGAGAAGGCCTTTGGTTCTTCCACAAAATTC
ATCGCTTCACTTAAAGAGAACTCCAACCAAGATCTTGTCTTGAAGGAATTC
ACAAACGATTTGTTACAAGAGGCTAGAAATTTGGCTACTTGCACTTATGAA
GCAAACACGCTATGGGGTGTTGAGGTAGGATTTTCGTATGATTGCTTGTTGG
AGAGTTCATACACTGGATATCTCTTACATTGTAAAGGATGGAAATCTGTGTA
TCTTTATCCAAAAAGAGCGTGCTTCTTGGGATGCACGACAATTGACATGAA
GGATGCGATTGTTCAATTAATAAAATGGACCTCCGGATTACTTGGAGTTGCC
ATGTCCAAGTTTAGCCCTCTCACTTATGCTATGTCCAGAATGTCTATTTTGCA
AAGCATGTGTTATGCGTACATCACATGTTCAGGTCTTCTTGCAGTTCCGCTC
TTTATATATGGTGTTGTTCTTCCATTCTCCCTACTTAGAGGCGTTCCTGTTTTT
CCCAAGGTATCGGATCCATGGATACTGGGTTTCGTGTTTGTATTTGTATCCTC
CCATGTTCAACATCTATACGAGGTGCTGTCAAGTGATCATTCAGTGCAACAA
TGGTGGAATGAGGTGAGAATCTGGATCATGAAAGCACTATCAGCCTGCTTG
TTTGGATCAACGGAAGCAATAATGAAGAAGATTGGGATACAGAAAACAAC
ATTCAGATTAACAAACAAGGTAGTGGAGAAAGAGAAGTTGGATAAATACG
AGAAGGGAAAGTTCGATTTCTCAGGGGCAGCAATGCTTATGGTTCCTCTCA
TCATTTTGACAATACTAAATTTGGTGTCGTTCATTGGAGGACTCATAAGGGT
GATCATCCACAACAACTATGATGATATGTTTGGCCAACTTTTCCTGTCCTTTT
ATCTCCTACTTCTTAGTTACCCTACTTTCGAAGGGATTGTTACAAAAGGTAC
AGACAAACTTAGGAAGAAAAAATAAGGAGTGATTGAATAACTGCCTAGTA
CAGTTTTCACTTTACTTCTCAAGATTAGTCCTCGTTTCTGTTTATTTTTATTAA
GATCCGCAACACTTGTAGCCGGCTGCATTCTCAATAATAATGAATTCAATAC
CATTTGTTTTTTCCCTCTTGCAAGAACAAAGAGTATACAAGTATATACAAAC
TTTTGATTATCA

>110724764 (*CqPER9*)
AGAGCAGGTAAAACTAGAGCACTAACATTAGTACCCTAAATGGAGCAGAG
GAACCACTTTGATATAGTCATATAAACAAAGTCATGTATCAAATTAGGAAAT
CTTGAGTTTAGTGGTTAGTTGTTTCTTCATAAATTTCACCACATTTTTAGTCT
ATAAATATCAACCAACTTTGTACCCTTAGAATCAACAATTCCAAACTCAAAA
ATCTTAGTAGAGTTTGATCAACACTTATGGCTACAAAAATGCACCTAGTGTT
TTTGGCTTTCCTTTCATTGATACCGCCTTTTATGGTTGCTCAAGCAGACCCG
GGTTATAGCTATGGGTACGGCTTTGGATGGGGTAGTGGTGGTGGGAACGAC
TATAGCTCGTTGTCTCCGTACTTCTATGACTTCTCTTGCCCTCAAGCTAACA
ACATTATTGCTTCGGTCTTAGAGAATGCTGTTGCACAAGACCCTCGGATGG
CTGCTTCTTTGCTTAGGCTTCATTTTCATGACTGCTTTGTTCAGGGGTGTGA
TGCTTCTATACTGCTAGATGATAGCGCGACATTTGTAAGTGAGAAAAAGGCT

AAACCAAATCTTAATTCCTTGAGAGGTTTTACTGTCATTGATGAAATTAAAG
CCAGGTTAGAAGAAGCATGCCCTGAAACTGTCTCTTGTGCTGATATCTTGG
CCTTGGTAGCTCGCGCCGCCAGTGTCTTGAGTGGAGGACCTAATTGGGAAG
TCCCATTAGGAAGAAGGGACTCAAAAGCAGCAAATATGCTACTTGCAAATG
CAACAATTCCTCCTCCTATTCTTACAGCCCAAAAGCTTATTGCATCATTTCA
ACAACAAGGCCTAGATGAAGTTGATCTTGTCGCGCTCTCAGGAGCACATAC
TATTGGGGTGGCGAGGTGTACAAGTTTTAAGCAAAGGTTGTATAGCCAAAA
TGGTAACAATCAACAACCAGATTTGAGCCTCGAAAAGGCTTACTTAAATGA
ATTGAAGTCAGTTTGTCCTTCATCAGGGGGTGACAACAACATAACTCCATT
GGATTATGCATCTCCAAAGACCTTTGATAACACTTACTATAAACTCATCTTG
GGAGGCAGGGGACTTTTGTTTACAGATCAACAACTTTATTCAGGAAATTAC
GCGAATTTAGCTCAATTGGTCAAGTCTTATGCTGAAGATGAAGGGTTGTTCT
TTGATCAATTTGCTAAGTCCATGGTTAAGATGGGAAAGATTAGCCCTCTCTT
AGGGTCTAATGGTGAAGTTAGGAAGTATTGTCGTCGCCCTAATTAATTCATT
TAAGTACTTTACCTCTTTAAGTAGGATGATTATTGGGTTTAATTTGTGATTTAT
TGGGTTCTTGTCAAGTTGTGAATCCGTACTAAGGTTTGATTATTCATGTTCG
ATTTAATTTTAAAAATTGTTGTTAATTTGTAAGGTTGATGTTAGTTGCAATCC
TTGTAATAATGAAATTCTTATGCAAAATAGTAAGGGTTGATTTAAGGGAGAT
ATGCATTGTACTGTGTTATAAGATTTATGCAATTGATCAATTATAACTTCTTC

>110735668 (*CqPER12*)
GCTTATAAATTGGCCTTTAAATTTAGCATTCGATACTACCTAGTGATCAAGAG
CAACAACAACTACTAAGTACTATAGTTAAGGCATTAAACTAGGTAACCAAA
AAACACAAAAAATGGGTTCCCAAATAAATTTGTTGTTCTTAATAATAATGTC
ATCTTTGGTAGTAGGAAGTTTAAGTAGTGTACCAGTTGCACCTGGACTATCG
TACTCATTTTATGGTTCAAAGTGCCCTCAATTAGAATCCATTGTTAGAAATCA
TCTTAAACAGGTTTTTCAGAAGGATATCACTCAGGCTGCTGGTTTGCTTCGC
CTTCATTTCCATGATTGCTTTGTTCAGGGATGTGATGGATCAGTATTGTTGGA
TGGGTCAGCAAGCGGTCCTAGTGAACAAGGCGCACCACCCAACTTGTCTC
TAAGGAAAGAGGCCTTTAAAATCATAAATGATATTCGTGCCCTTGTCCATAA
AACATGTGGTCGGATTGTTTCTTGCGCAGATATCACTGCACTTGCAGCTCGT
GATTCTGTTGTTCTGTCTGGTGGACCAAATTACAACATTCCACTAGGAAGG
AGGGACAGCCTAAACTTTGCAACAACACAAGTAACACTAGATAATCTACCG
GCACCTTCTAGCAGCGCCTCAGCCATTCTAAAATCCCTTGGCAACAAAAGG
TTTGATCCTACTGATGTTGTTGCCCTTTCCGGAGGCCACACCATTGGTCGCG
GCCATTGTTCCTCCTTCACCGACAGGCTCTACCCTACACAAGATAAAACAA
TGGACCAAACTTTTGCTAAGAACCTTAAGGCCACTTGCCCGGCCAAAGAC
ACTGATCGCACCACAAATTTGGACATCCGTACTCCTAATGTATTCGACAACA
AATACTACGTAGACCTAATGAATCGCCAAGGCTTATTTACTTCAGATCAAGA
TTTGTACACTAATCCAATTACAAAGCCGATTGTCACAAGCTTTGCGACAAAT
CAAAATTTATTTTACGAAAAATTTGTCGTTGCGATGTTGAAAATGGGACAA
CTTGGTGTGTTGACGGGTACACAAGGTGAAATTCGTGCAAATTGCTCGGCA
AGAAATGCTAAGAAGGTAGACATGTGGTCTATCATTGACGAGGGTATCACA

ATTTTAAGTGATATGTAATTAACTAGCTATATTTTGAGTTGATTGCAATAACTT
GTGAGGTTTAATTAGATGTTTTGTCGTTAGTTTTTAAGGCTTCATACTTCTAT
GACAAATAAAATGTTTGACTAGGTAAAATACTCGTACAATTTTATCTAGATC
AGATCTAGTAGCATGCGTAATAATAGAAGTATTGTACTTGAACTTGATCTTTG
TCTCTTTTATCAAATTGTCACTAATTTTAATTAA

>110712995 (*CqACO1*)
TTATCTCTAGGACTAATAAGATAACTAAAGAGAGAAAATGGAGATACCAAC
CATAAACTTTGGTCAGCTTGAAGGTGAGAAAAGAGGTGAAACAATGGCAC
TTTTACATCATGCCTGTGAAAAATGGGGATTTTTCCAAATAGAAAATCATGG
CATAGACAAGGAGTTGATGGAAAAAGTGAAGCAATTCATCAATATATATTAT
GAAGAAAAATTGAAAGATGGCTTCTTCAAGTCAGATATTAGCAAGGCTTTG
GAGAACAAGGAGAATGCTTCTAACAGAGATTGGGAAAGCAGCTTCTTCAT
TTGGCATCGCCCCGAGTCTAACATAAATAAATTTGCTGATTTCTCTGAAGAT
TTCCGGAAAACGATGGAGATTTACGTCAATCAGCTAGTTAAGGTTGCTGAA
AAACTATCCGAGCTAATGTGTGAAAATCTTGGACTAGAAAAAGACCACATA
AAGAAAACATTCTCAGGAATCAAAGGCCCTGCAGTAGGAACAAAAGTGGC
TAAATACCCTGAATGTCCGAACCCTGAACTTGTCAGAGGCCTCCGCGAGCA
CACTGATGCAGGCGGAATCATTCTGTTACTGCAAGATGATCAAGTTCCAGG
CCTCGAGTTTTTCAAAGATGGTAAATGGTTTAAGATACCGCCTTCTAAGAAC
AACACGATCTTTATCAATACAGGGGATCAGATCGAAGTGCTTAGCAATGGA
AGGTATCAGAGCATTCTGCATCGCGTTATGACAGGAAAATGTGGAAGCAGA
TTGTCTGTTGCTACGTTTTACAATCCTTTTGGGCTTTCTGACTTTGTTCCTGG
ACGGGTAGTGACTGATGCGGCTAATCGGAAGCGGGTGAAGTATGATTCTAG
TTGTCGAGCAATAGGATATGGCTTCCTTCCTTTCTCTTTCTCTTCTCTTGGGG
AGTTAGAAAAGGAGTCCAATGAATTTGTGACCACTTTTCGCATATTCCGAA
AATTACAAGCTACTTATATTAACAAATAA

>110699138 (*CqCPA*)
AAAAATGTATGTACTAAAATTATAAACAAATTTTTTATATTGTATTTATTGCAA
CTTTGCTCCATTTTCTAAATTTTATTTTGAAGTCCTATGGACGGAGAGCGTG
ACATATTCTTCCGTAATTGTGCTTTCAAAGTCTCAAAGATGCACAAAATTTC
ACTATATATATATAACATCCAAACTTCATACTTTCAATACATAAAACTTTTCCA
ACATAGTCTTTTCGCAATCAACTTAAACAACAATAATAATAATGTTAAACAT
AACAGCCATCAAAACTGCCTCTGATGGCATTTTACAAGGTGATAATGCCCTT
CAAACCGCCTTCCCCTTACTCATTATACAAACTACCTTGATCCTCGTCGTAA
CCCGCCTCCTCGCTTTCGCCTTCAAGCCTCTCCGACAACCCAAAGTCATTG
CTGAAATTGTGGGAGGTATATTGCTGGGACCATCCGCATTTGGCAGGAACC
AACAATACATGGATAAAATCTTTCCAAAATGGAGCACACCAATTTTAGAATC
AGTTGCTAGCATTGGTCTTCTTTTCTTCTTATTCCTAGTAGGCCTTGAGTTGG
ACCTAGGCTCTATTCGTCGTAGTGGCCGTGAGGCCTTCACCATTGCTCTAGC
CGGAATAAGTCTCCCCTTTATCGGTGGTGCGGCGGTAGCTGTTATACTCCGC
CGCACCATCGAGGGAGCTGACCGAGCTGGTTACGGTCCTTTCCTTGTGTTC
ATGGGAGTTTCACTCTCTATCACCGCCTTCCCGGTTTTAGCCCGTATTTTGG

CTGAACTCAAACTCTTGACCACCAAAGTAGGGGAGACAGCCATGGCAGCG
GCCGCTTTTAATGATGTAGCTGCATGGATCCTCCTTGCTCTCGCAGTTGCTC
TTGCAGGCAACGGCGAGGCAGGTGGTCATAAAAGCAGCCCGTTAGTCTCG
TTGTGGGTGTTACTAGCCGGGGTTGGGTTCGTAGCATTCATGTTCTTGGCTG
TTAAGCCCTTAATGGCCTGGATGGTCCGACGTTGCTCAGCAGAGCAGGGTG
TGGTGGACGAGCTTTATATCACTATAACCCTAGCCGGTGTACTCGTGGCCGG
GTTCATGACCGACTTTATAGGCATTCACTCCATATTTGGAGCATTCATTTTCG
GTTTGATTATCCCTAAAGAAGGTGATTTTGCCGAGAAGCTACTAGAAAGAA
TTGAGGATTTTGTTTCAGGCCTATTGTTGCCACTGTACTTTGCCTCTAGCGG
TCTAAAGACCGACGTCACAAAGATCCAAGGGGCCGTCGCGTGGGGTTTAC
TCGTGCTGGTGATCGCGGTCGCGTGCTTTGGCAAGATTTTTGGTACCTTTGT
GGTGGCTAAGTTTTGTAAAATGAAGGCTAGGGATGCGTTAGTCCTTGGTGT
GTTGATGAACACAAAAGGTCTGGTCGAGCTCATTGTCCTCAATATCGGAAA
AGAAAAAAAGGTTCTCAATGATGAGATGTTTGCAATTTTAGTGCTAATGGC
ACTCTTCACAACCTTTATAACAACACCAATAGTAATGGCAATCTTCAAGCCA
GACAGTTTTGAAACCCAAAAGAGGAAGCTTCAACCTGATGCATCTGTACAT
ATGGACTCTAGCACTACACCGGCAACCTCGGCTTTTAAAGAACAGTTCAGA
GTCTTGGCCTGTGCTCATGGTCCAGGGAACGTATCCGGCCTAATAAACCTCA
TCGAATCAACCCGAAGCACGGCCTCGAAGAACAATCTTAAACTCTACATTA
TGCACCTTGTAGAGCTCACTGAACGTTCTTCATCTATTCTCATGGTCCACCG
CCTTCGTCGTAATGGGTTCCCGTTCATTGACCCAACTCGACGTCGTAGTCA
AGTTGAGGTACATGACCGTATAGCTTCGGCTTTTGAGACCTACGCTCAATTA
GGCAGGGTTATGGTTCGTCCTGTGACTACTATATCAGCCCTTTCGACGATGC
ACGAAGATGTTCGACACGTGGCAGAGGGAAAGAGGGTGATGATGATCATA
ATGCCATTTCATAGGATGTGGAGGAAGGATGAAGAAGGGGAGATGAAATTG
GTGGAGAATTTAGGGCATGGATGGAGAGGTGTGAACCAAAAGTTGCTAAA
GAATGCTCCTTGCACGGTTTCGGTATTCGTGGACCGGGGTTTAGGTGCGGG
TCGTGTAGCTCAGGATGATGCACAAAGGCAGCTTAGGGTTTGTGTTATATTC
TTTGGAGGGGCTGATGATCGAGAGGCTTTGGAGCTTGCTAGAATGATGGAC
CATCCCGAAATTCGGGTTACGATTATAAGGTTTGTAGAGAGCCAAGGAAGG
GATGCTAACAATGTCAAGCTAATGCCTTCCCCTGAAAAGTGTAGTGATAGTA
ATTACACATTCTCTGTTGCTGCCATGAATCGAACGGAGGAAACGGCACTTG
ATGAGGCAATGGTGGAAAACTTCCGCAACAAATGGGATGGAACAATGGAA
TTCATACAAAAACAAGCTAACAACATAGAGGAAGCAGTGTTGACACTTGG
TCGAAGTGGTGAGTACGACCTTATAGTCGTAGGCAAAGGTCGTTTCCCTTC
GACGATGGTGGCCGAACTAGCTGACCGCCAAGCCGAGCATGCCGAGCTAG
GGCCTATAGGGGACATCTTATCATCAACTAACAATGGCATTGTGTCATCTGT
GTTGGTTATTCAACAACATGCTTCGGCACATGTAGAAGAGATGCCTGTTGAT
AAAGTAGTTGAAGGTGATGTTAATGTCCAAGATGCTGCTAACCAAGTTTGA
TAATCTTTTGGCACAAAGTCTACAACATGGTAAATAGATTTTGTAATACGTTT
AGTGTTGTACTGTGCACTGTACAAAATTTGTGTCTCATTTCTTGTACTTTAA
ATATATAATTATAATATGAAATATACGGAGTACCATAATCTCTCA

>110734631 (*CqPK*)

ATGGGTTTGGGACGCTTTACATACATACTTCTTTTAGTTTTTACTTTTTCAAT
ACTAATTTTCGATTTTTTCGTGGTCGCTAATTCTAACCAGCCTTCCTTGCAGC
AATCGAAGCCATTGTGCCATGACCAAGAGAAGATTGCATTGCTTCATTTTAA
GCAAAGCTTTCTTCTTGATTGCTCCACGTCATCATTTTCTGATTTCGCATATC
CAAAGGTGAAATCATGGCATGATGAATTAGATATTCATCATCCGGAAAATGC
CACTGTAAGTGGTGATTGCTGTAGGTGGGATGGTGTCAAGTGCAATGAGGA
GACGGGTCACGTGATTAGCCTTAACCTTAGTAGCAGTTGCCTTTACGGCAC
CTTCCCCCGTAACAGCACACTCTTCAGCCTTAATCATCTCCGTGAGCTTGAT
CTTTCTTTTAATAACTTCAATTATTCTCAAATCCCCTCTGACATTGGTCATCTA
TACAAGCTCACACACCTCAACCTCTCCTATTCAGTTTTAAGTGGTCAAATTC
CACCAGAAATTTCAAAACTGTCAAGCCTATCTCTTCTTGATCTTTCCTTTAA
TGGAGATCGGCCTACACGTGTTAATGAGAAGTTAAATCTGAAACTTAATGA
TTTGAGTCTAGAGAAGTTGGTCTATAATTTAAGTCGTTTAACTCATCTTCATC
TAGACTTAGTTGACGTATCGTCTGAGGTACCCCTTAGCTTAAGCAACCGAA
CTTCCCTAAAAGCTATCTCCCTCCCTTTCTGCAATCTATTGGGTGAATTGCC
ACAAAGTATTTTCTGGCTACCAAAACTTGAAGAAATTTGGTTGGGTTTCAA
TTCTAAATTAGGAGGATATCTTCCTGATTTTCATTCTAATAGTCCTCTTCGTG
CTTTAAGTCTATGGGAAACAAAATTTTCAGGGGAATTGCCAGATTCACTTG
GAAATCTGGTTTTCTTGAATATTATGATTCTGGAAGATTGTGAATTCTCTGG
AAAAATTCCATCCTCAATAGGTAATTTGACAGACCTTACCATACTCGGCCTT
GCTGGCAATTATTTTAGTGAGCTCCCTCACTCTATCATGAACCTAACACGTC
TTACTTCTTTATCCCTTTCAAACATGATGAATGTTGAGACTGCTGGAATATTG
AATTCTTGGTTGCTTAAGCTAAATTTTATCACTCATCTATACCTTCCCCATATG
AATTTAGGAAATGAATTTTTTCCTACTCTGTCTAACTTTACCAGACTCTATGA
TTTGGAGCTTAGCAACAACTTGTTAACAGGCCCACTTCCAAGTTGGCTCAT
GAATCTAACCCAATTAGAATCCTTAGACCTATATATATGGTAA

>110725220 (*CqFK*)
ACAAATAGTAAAAGTATTCTTCTTTAATTAAAAGAAAGAAACAACCCTTAA
ACGTCGTACGAGTATGACTTGTATGAGTGAATGATTCCGAATAACAGATATA
AATTAAGATCAGGGTCGTATACAATTCACAAAAATATTGGTATAAGTTTTCT
GTAAAAAAAAAAAAAAAAAAAAGTGCAGGCCTCTGTTCTTTGTCGTCAAT
TTCCTTCACTGGAAAAATGAGAATGAAAAGAAAATCCCTAATTGAGGAAA
GGTATTACGGGATCAAAAAGAGGCAGATGAGTTTTATAAAGATGGTACAAG
AAAGAGCTGTTAAAATGAAAACTTCTTGGGTGCCTTTTTTACCTAATGATTT
GATGTTTAAGGTCTTTCTTTTATTACCAATCAAATCTCTTTTGAGGTTTACTT
GTGTTTGCAAGGCATGGTATAAGTTGATCCATTGTGCTGAGTTTGTTGAGGC
TTACAATAATCATGCTGAGACCACCCCTATCATACTCCGGGGAGTTGATAAC
AAAAGACCGTTCACATTCCATGTCGAAACCCAACTCAGCCAAGCTGAAAG
CTTTTGTTTGTTTCCTTGTAGCTCGGGGACTAAACAAAGCAAGTCCATCCAT
TTCTTGGAAATTGACAATGAGAAGGGCAAGTTAATTGACCTCAATATTAGCT
GTTCCGGATCACTTGTATCCACTTGCAATGGGTTGATTTTGATCACCTCTATG
CAAGGACTTATGAAGGAAAGAAATTCCTTTTTGGATCAGCCTTTGGTTGGC
AGTCAAGAAAAACCAGGGCGGTTAATTGTGATGAATCCTATGACGAGAAA

ATTGATAGGGTTTCCTCCAGGAACTCTTCCGAGTAAACTTCATGAGGAATC
GTATGGGCTAGTTTATAGCCATTTAGAAGGTGTTTTTAAAGTGGTGCATTTG
TTCAAGGATAAATCAGGGTGTATTGCTTGTGAGATTTTAAGCTTGCAGACA
AGATCATGGAAAGCGGTTAATGGGCCAGTTGGAACGCTTTTTAATAAATTA
GGCCAGGCACCTATTTCAGCTCTTGGAGCTTTGCACTGGCTTCCTGGTCCAT
CTAATTCCAATTATATAATCTCTATGGGGGCTGATGATGAAAAATTCTCCGTG
ACAGACCTTCCAATGACCATGGGAATGTATGATAGGCTAGTCGAAATGGGT
GGTTTTCTGAGCTTTGTGAACAGTTTGGATATGGACCATATAGATGTGTGGG
TCTTGAAGGGGTTAGAAGGAACGAAGTGGGTAAAGCAGCACACCATTTGC
ATCGATGCTCTTTTGGGTTATGTGGATAATGAGGAGTACTCTTTACCTTCTTT
TGGTTTGAATGCCAAAGAAATGGTATTTAGGAGAAAAAAGAGATTGTATTC
TTACGACTTTGAGCTTGAAGAAATCAGAGAGATTGAAATGGACCATGAAA
GTATCACAGCATATGAGAATATTATACCCCACTCGAATAACCTTGCTACATGG
GAATCTCTTGAACCAATGAGTTAATCCTGAAGATTTTTTGCACCATGCCTTG
AAGTTCTTGAAGGCCTGAAGCTGCTCGAGAACATCTGAACATGCGTTAAGC
AAACCGTGTTCATTATGTTCCTTTCCACTCACATATATATTCATAATCTATTTG
AAACTTTGGATCAGCTCGATGACTTGATTTTATGGTCGTGGTTTGATTATAG
GTATGCTGCCATATGCAGTCCACATATATACACTCTTGTATATTTGGAAGTTG
TTGCACTAAGAGATTGAAGTATTTATGCAGATATAGAGCTTGTTCCAGTGAT
CTAAAATTGTGTTTAAGGCGCATCTTTTACCTTCTATTTTGCGGGTTTGTGCT
AACACGATCTTTGCTGTTTGTACTTCCTCCGTTTTTTTAGTTGATACCATTTG
ACTTTTACACTATTCA

>110735797 (*CqPDP*)
CTAACTTATTCAACTCAAACATAAATAATTAAAAAAAAAAAAAAAAAAAGT
TCCCCAAGTCATCTTCTCCTATCCTCCTTCCTTCCCCACTAAATCTTAGAAG
AAAAAAAATGGGTTCTCTTCCTGAGACCAATGGTAATGCAGTCGTGGGGA
GGACAACCGTGCATCGTTTGATCGCGGAGGAAGGATCGGTGTTGATGCCG
GGTATCCAAGACGCTTTCTCTGCCGCCATTTGTGCCAAAACCGGCTTCAAA
GCTTGCTTTGTCTCCGGTTTTGGTGTTTCCGCTGCCCTCCTCGGCTTGCCTG
ACTTTGGCTTGCTTACGACCGCAGAAGTTGTAGGAGTTGTCCGTAGAATGA
CTGCTGCTGCTCCAAGTTTGTGTGTCGTTGTTGATGGAgACACCGGAGGTG
GTGGCCCTCTCAATGTGCAAAGATTCATCAAAGACTTGATTGCAGCCGGTG
CTAAGGGTGTTTTCCTAGAGgATCAAGTATGGCCAAAAAAGTGTGgTCATAT
GCGTGGTAAGAGTGTTGTACCTGCAGAAGAGCATGCACTTAAAATTGCAGC
AGCAAGAGAAGCTATTGGTGAATCTGATTTCTTCTTGGTAGCAAGAACTGA
TGCTAGAGCACCACACGGTCTTCAAGAAGCAATTAGACGTGCCAACCTTTA
CAGAGAGGCCGGAGCTGATGCAACCTTCGTTGAGGCACCTGCAAACATCG
ATGAGCTTAACGAGGTTGTCAAGGGTACAAAAGGTTTGAGAATCGCTAACA
TGATTGAAGGTGGAAAGACCCCATTACATACACCAGCAGAGTTCAAGGAG
ATGGGATTCCACTTGATTGCTCATTCACTATCAACAATCTATGCTACAACCA
AAGCTTTGGTTGGAATCATGAAGGTTCTTAAGGAAAAGGGTACCACTAGGG
ATGACTTGGACAAGATGGTTACTTTCTCTGAATTTAATGACATGATTAGCTT
GGAATCATGGTATGAAATGGAATCAAAGTTCAAGAACTTCACCCCAAAATC

TTTGGAATCCTAAGACTTTTTACCTATCTTGGCATGTTTAGCTCGACATATAC
AAGAGTTGAAATTAAAGACATGCATGTTTGTGCAATTGAGTTTTGTAAGATT
ATGAAATTAGTTTAATATTTGTTTGAGAAAATAATAATTTTGCATATTGAGTA
ATGTTGTAAGTTTATTTAAAACCTGTTTTGAGAGCCTGCAAGGTGTAGATGC
TTGTAATCTCATCAATATTAGTAATCAAATATAATTCGAAGCTATTTTTTCTA

>110724665 (CqACTIN)
AAGGATAGCAAGTCACTCATTCAATCGCCTCTCTCTCAAAATTCTCGCTTTC
TCTCTCTAAATTCTCTCTCTAATCTCTTTCACTTTCTCTCTCTACTTTCTCTCT
CCTCTCGCAGCCGCAAGCTCGCTTCGTTCTTCAAGTATATAAATCGAGATGG
CTGATGCAGAGGAGATCCAACCCCTTGTCTGTGACAATGGAACTGGTATGG
TCAAGGCTGGATTTGCCGGTGATGATGCTCCAAGGGCCGTCTTCCCAAGTA
TTGTTGGTCGTCCCAGACACACTGGTGTCATGGTTGGTATGGGACAGAAGG
ATGCATATGTTGGAGATGAAGCTCAGTCCAAGAGAGGAATTTTGACCTTGA
AGTACCCAATTGAGCACGGTATTGTGAGCAACTGGGATGATATGGAGAAGA
TTTGGCATCACACCTTCTACAATGAGCTTCGTGTTGCCCCAGAAGAACACC
CAGTGTTGCTTACTGAGGCACCATTGAACCCTAAGGCTAACAGAGAGAAG
ATGACCCAGATCATGTTTGAGACCTTCAATGTGCCCGCTATGTATGTTGCCA
TCCAGGCCGTTCTATCTCTGTACGCTAGTGGTCGTACCACAGGTATCGTGCT
TGACTCCGGTGATGGTGTGAGTCACACAGTTCCCATTTACGAGGGTTATGC
CCTCCCCCATGCCATCCTCCGTTTGGATCTTGCCGGACGTGATCTTACCGAT
TATCTCATGAAGATCCTTACCGAGAGAGGTTACATGTTCACAACATCAGCTG
AACGGGAAATTGTCCGTGATATTAAGGAGAAGCTTGCATACGTTGCTCTTG
ACTTTGAGCAGGAATCAGAGACTGCCAAGAGCAGCTCTGCCATTGAAAAG
AACTACGAGCTTCCTGATGGTCAGGTCATTACCATTGGAGCTGAGAGATTC
CGTTGCCCAGAGGTCCTCTTCCAGCCATCATTGATCGGTATGGAAGCTGCA
GGTATCCACGAGACTACCTACAACTCCATCATGAAGTGTGATGTTGATATCA
GGAAGGACCTATACGGTAACATTGTGCTCAGTGGTGGTACAACTATGTTCC
CTGGCATTGCTGACCGTATGAGCAAAGAAATCACAGCTCTTGCTCCCAGCA
GCATGAAGATCAAGGTTGTTGCACCTCCTGAGAGGAAGTACAGTGTCTGGA
TAGGAGGGTCTATCCTTGCGTCTCTCAGCACCTTCCAACAGATGTGGATCTC
CAAGGGAGAATACGATGAATCCGGCCCATCAATTGTTCACCGAAAGTGCTT
CTAAGAAGATAAGTTTTGAGATTGTAGTCCTACATAGTCTCCTGTTGTCTCT
TTGTTTTTCTTTCTGTTTGGTCAAAAAAAGAAAAAAAAACGTATTAGTGTG
GTGTATGATTTGATTTGATTTGGTCGAGAGAGATGGAGAGGGTTTAGGAGC
TTGGAATTTCTATTGTTTGTTGTAACTATTTTTCGAGAATTATGGTATGTAATC
TTTGCGGATTGCAGAATGTTTAATTTCTATACTCTACTATTTTTCTGGGTCA
