# Supplementary material for: Genome-Wide Transcriptomic and Proteomic Exploration of Molecular Regulations in Quinoa Responses to Ethylene and Salt Stress
Source: Plants (Basel). 2021 Oct 25;10(11):2281. doi: 10.3390/plants10112281 (PMC8625574; doi:10.3390/plants10112281)
Supplement: Supplementary file 1 [file plants-10-02281-s001.zip › plants-1403961/SUPPLEMENTARY MATERIALS/SUPPLEMENTARY MATERIAL TABLE S2-QIAN MA.pdf]

Supplementary material 2: Oligonucleotide primers used in qRT-PCR confirmation.

Gene ID	Annotation	Description	Primer Name	Primer Sequence (5'→3')
110717159	*CqGLC*	glucan endo-1,3-beta-glucosidase	CqGLC-Fq	AATCATGCCTGGTGAGGCCG
110717159	*CqGLC*	glucan endo-1,3-beta-glucosidase	CqGLC-Rq	AAACACCGGCAGAAGGTGGG
110722212	*CqABCB*	ABC transporter B family member 25	CqABCB-Fq	TTTGGTGCCATCCGCACAGT
110722212	*CqABCB*	ABC transporter B family member 25	CqABCB-Rq	GCACCAGGTGTCATGCCAGT
110688100	*CqNRT2.1*	high-affinity nitrate transporter 2.1	CqNRT2.1-Fq	TGCACATTGCCCGTGACCTT
110688100	*CqNRT2.1*	high-affinity nitrate transporter 2.1	CqNRT2.1-Rq	GCAGACTTCCCACCTCGCTC
110711362	*CqAOBG*	anthocyanin 3'-O-beta-glucosyltransferase	CqAOBG-Fq	GACCACTGAACTCGCTGCCA
110711362	*CqAOBG*	anthocyanin 3'-O-beta-glucosyltransferase	CqAOBG-Rq	TTGGATGTGGGAGTCCCGGT
110717430	*CqCSI*	cellulose synthase-like protein G2	CqCSI-Fq	TGGCGGCAACACACATTTGC
110717430	*CqCSI*	cellulose synthase-like protein G2	CqCSI-Rq	AAACGAGGTCGGCGATGGTC
110724764	*CqPER9*	peroxidase 9-like	CqPER9-Fq	GGGTACGGCTTTGGATGGGG
110724764	*CqPER9*	peroxidase 9-like	CqPER9-Rq	GAAGCAGCCATCCGAGGGTC
110735668	*CqPER12*	peroxidase 12-like	CqPER12-Fq	TTGGATGGGTCAGCAAGCGG
110735668	*CqPER12*	peroxidase 12-like	CqPER12-Rq	GAATGGCTGAGGCGCTGCTA
110712995	*CqACO1*	1-aminocyclopropane-1-carboxylate oxidase 1	CqACO1-Fq	GGCATCGCCCCGAGTCTAAC
110712995	*CqACO1*	1-aminocyclopropane-1-carboxylate oxidase 1	CqACO1-Rq	GCGGAGGCCTCTGACAAGTT
110699138	*CqCPA*	cation/H^+ antiporter 20-like	CqCPA-Fq	GCACCATCGAGGGAGCTGAC
110699138	*CqCPA*	cation/H^+ antiporter 20-like	CqCPA-Rq	TACGAACCCAACCCCGGCTA
110734631	*CqPK*	serine/threonine-protein kinase	CqPK-Fq	TCCTTGCAGCAATCGAAGCCA
110734631	*CqPK*	serine/threonine-protein kinase	CqPK-Rq	TACGGGGGAAGGTGCCGTAA
110725220	*CqFK*	F-box/kelch-repeat protein At3g23880-like	CqFK-Fq	TGGATCAGCCTTTGGTTGGCA
110725220	*CqFK*	F-box/kelch-repeat protein At3g23880-like	CqFK-Rq	AAGCGTTCCAACTGGCCCAT
110735797	*CqPDP*	petal death protein-like	CqPDP-Fq	TTGGCTTGCTTACGACCGCA

110735797	*CqPDP*	petal death protein-like	CqPDP-Rq	AGCACCGGCTGCAATCAAGT
110724665	*CqACTIN*	actin	CqACTIN-Fq	TGAGCTTCGTGTTGCCCCAG
110724665	*CqACTIN*	actin	CqACTIN-Rq	GGCATGGGGGAGGGCATAAC
