# Supplementary figures and images for: Genome-Wide Transcriptomic and Proteomic Exploration of Molecular Regulations in Quinoa Responses to Ethylene and Salt Stress
Source: Plants (Basel). 2021 Oct 25;10(11):2281. doi: 10.3390/plants10112281 (PMC8625574; doi:10.3390/plants10112281)

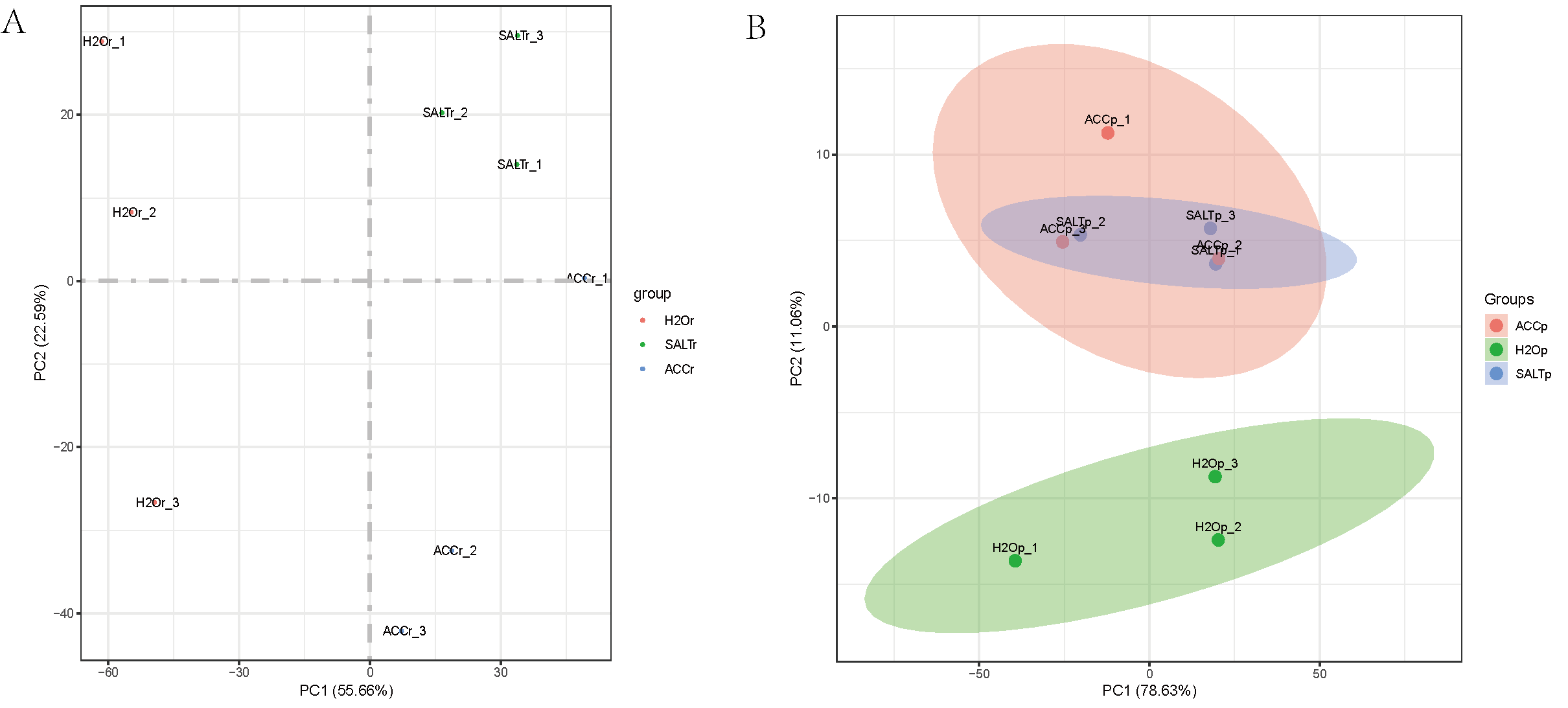

Supplement: Supplementary file 1 [file plants-10-02281-s001.zip › plants-1403961/SUPPLEMENTARY MATERIALS/SUPPLEMENTARY MATERIAL FIGURE S1-QIAN MA.png]

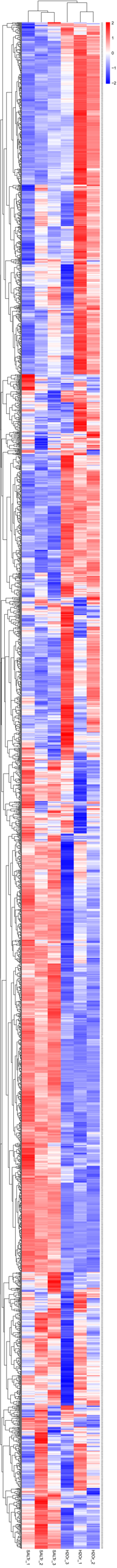

Supplement: Supplementary file 1 [file plants-10-02281-s001.zip › plants-1403961/SUPPLEMENTARY MATERIALS/SUPPLEMENTARY MATERIAL FIGURE S2-QIAN MA.pdf]

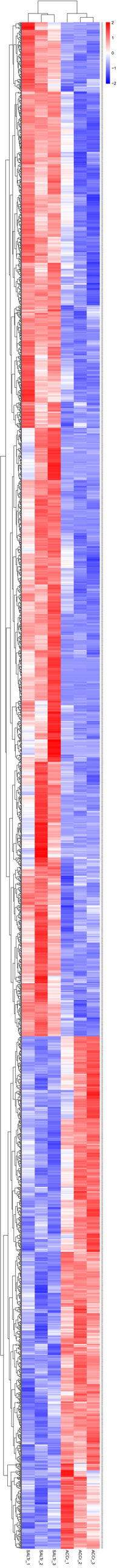

Supplement: Supplementary file 1 [file plants-10-02281-s001.zip › plants-1403961/SUPPLEMENTARY MATERIALS/SUPPLEMENTARY MATERIAL FIGURE S3-QIAN MA.pdf]

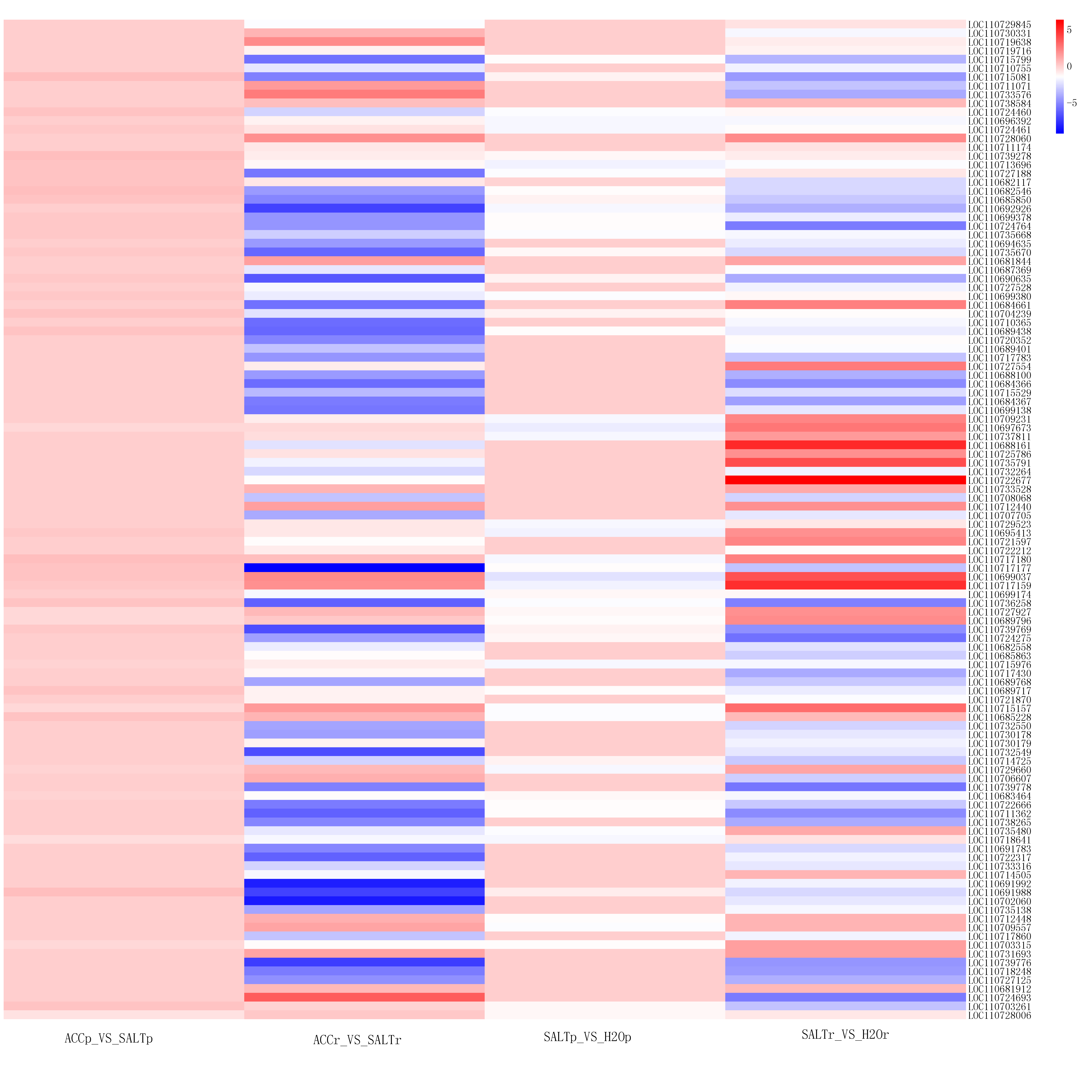

Supplement: Supplementary file 1 [file plants-10-02281-s001.zip › plants-1403961/SUPPLEMENTARY MATERIALS/SUPPLEMENTARY MATERIAL FIGURE S5-QIAN MA.png]

SALTp.vs.H2Op

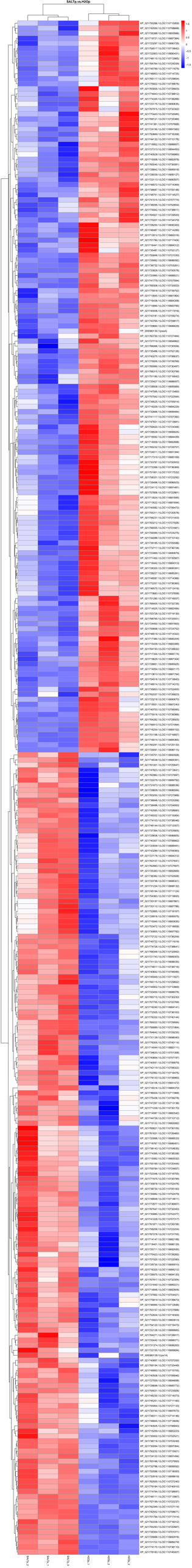

Supplement: Supplementary file 1 [file plants-10-02281-s001.zip › plants-1403961/SUPPLEMENTARY MATERIALS/SUPPLEMENTARY MATERIAL FIGURE S6-QIAN MA.pdf]

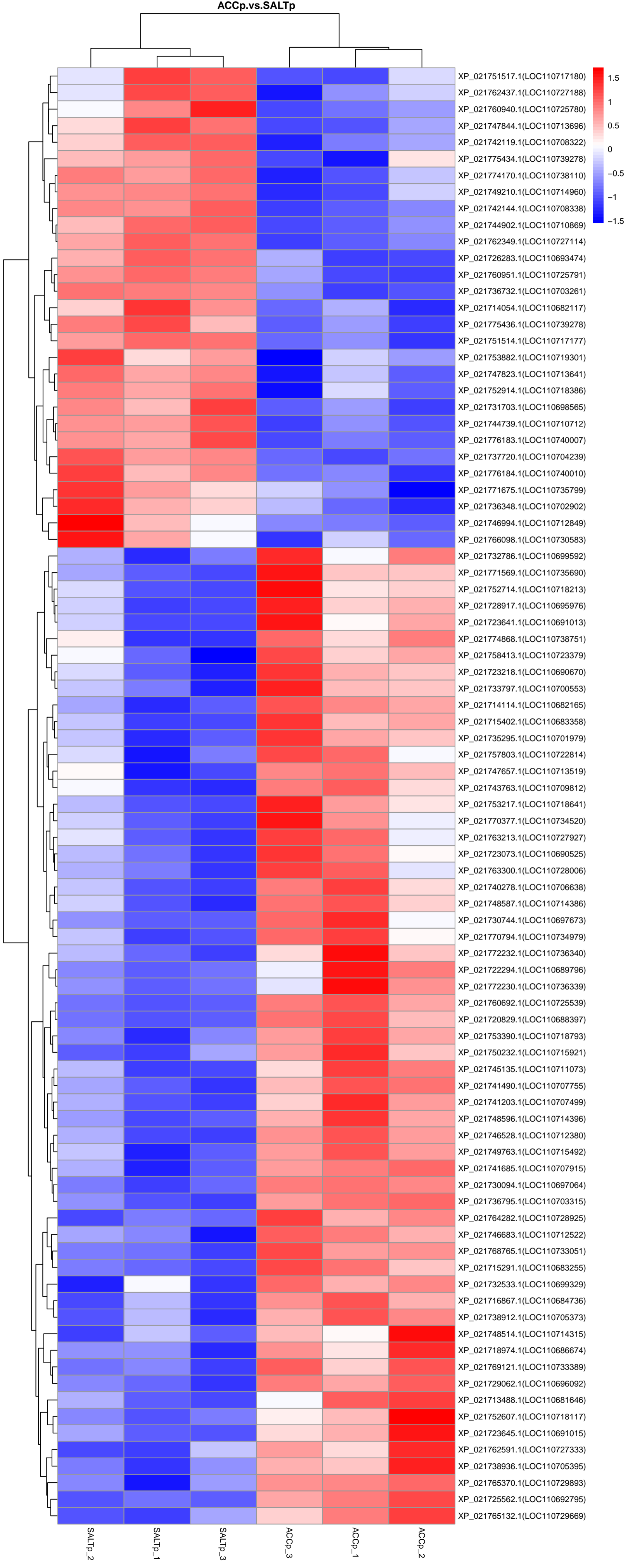

Supplement: Supplementary file 1 [file plants-10-02281-s001.zip › plants-1403961/SUPPLEMENTARY MATERIALS/SUPPLEMENTARY MATERIAL FIGURE S7-QIAN MA.pdf]

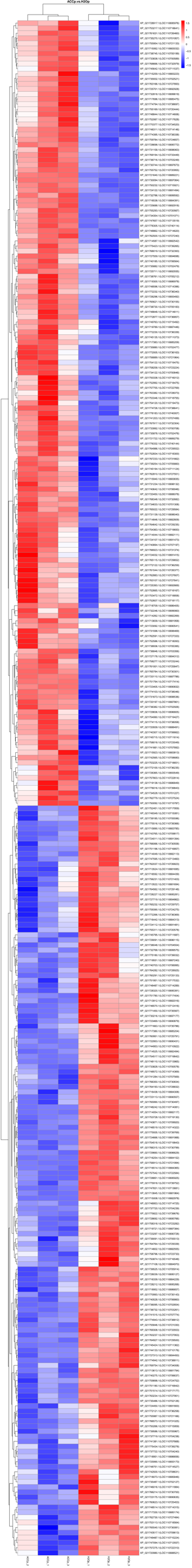

Supplement: Supplementary file 1 [file plants-10-02281-s001.zip › plants-1403961/SUPPLEMENTARY MATERIALS/SUPPLEMENTARY MATERIAL FIGURE S8-QIAN MA.pdf]
