# Supplementary material for: Genome-Wide Transcriptomic and Proteomic Exploration of Molecular Regulations in Quinoa Responses to Ethylene and Salt Stress
Source: Plants (Basel). 2021 Oct 25;10(11):2281. doi: 10.3390/plants10112281 (PMC8625574; doi:10.3390/plants10112281)

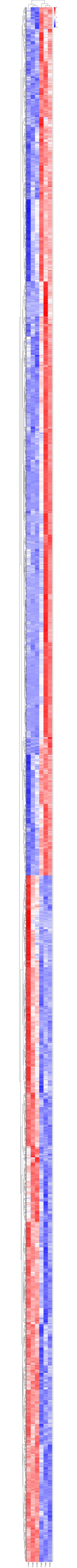

Supplement: Supplementary file 1 [file SUPPLEMENTARY MATERIAL FIGURE S4-QIAN MA.pdf › plants-1403961/SUPPLEMENTARY MATERIALS/SUPPLEMENTARY MATERIAL FIGURE S4-QIAN MA.pdf]
